# Is Retinal Microvascular Abnormalities an Independent Risk Factor of Vertebral Fractures? A Prospective Study From a Chinese Population

**DOI:** 10.1002/jbm4.10017

**Published:** 2017-10-10

**Authors:** Junping Wen, Kaka Tang, Fengye Zhu, Wei Lin, Huiying Rao, Huibin Huang, Jin Yao, Ling Chen, Jixing Liang, Lixiang Lin, Hongjie Chen, Meizhi Li, Xueying Gong, Shushan Peng, Jieli Lu, Yufang Bi, Weiqing Wang, Guang Ning, Pengli Zhu, Gang Chen

**Affiliations:** ^1^ Department of Endocrinology Fujian Provincial Hospital Key Laboratory of Endocrinology Fujian Medical University Fuzhou China; ^2^ Department of Ophthalmology Fujian Provincial Hospital Fujian Medical University Fuzhou China; ^3^ Department of Endocrinology Ruijin Hospital Shanghai Jiaotong University School of Medicine Shanghai China; ^4^ Department of Geriatrics Fujian Provincial Hospital Key Laboratory of Endocrinology Fujian Medical University Fuzhou China

**Keywords:** OSTEOPOROSIS, GENERAL POPULATION STUDIES, BONE MINERAL DENSITY (BMD), RETINAL MICROVASCULAR ABNORMALITIES, VERTEBRAL FRACTURE

## Abstract

Low bone mineral density (BMD) and microvascular diseases (MVD) share various common risk factors; however, whether MVD is an independent risk factor of vertebral fractures is incompletely understood. The aim of this study is to clarify whether MVD is an independent risk factor of vertebral fractures. In this prospective study, calcaneal BMD and retinal microvascular abnormalities were assessed at baseline from June 2011 to January 2012. A total of 2176 premenopausal women, 2633 postmenopausal women, 2998 men aged <65 years, and 737 men aged ≥65 were included. Then with/without retinal microvascular abnormalities cohorts were followed for an average of 2.93 years to find out the relationship between MVD and vertebral fractures. At the baseline, after full adjustment, retinal microvascular abnormalities were related to risk of low BMD only in men aged ≥65 years (odds ratio [OR] = 2.506; 95% confidence interval [CI] 1.454–4.321; *p* = 0.001). After follow‐up of 2.93 years, retinal microvascular abnormalities were related to risk of vertebral fractures in men aged ≥65 years (OR = 2.475; 95% CI 1.085–5.646; *p* = 0.031) when adjustment for confounding factors. However, no associations were found between MVD and vertebral fractures in men aged <65 years, premenopausal women, and postmenopausal women. When stratified by diabetes, in the without‐diabetes group, the men with retinal microvascular abnormalities had higher risk for vertebral fractures than without retinopathy (OR = 2.194; 95% CI 1.097–4.389; *p* = 0.026); however, the difference was not found in women. In the diabetes group, there were no significant differences of risk for vertebral fractures between those with retinal microvascular abnormalities and those without both in men and women. Stratified by hypertension, the men with retinopathy had higher risk for vertebral fractures than those without among the hypertension group (OR = 2.034; 95% CI 1.163–3.559; *p* = 0.013), but a difference was not found among women. In the without‐hypertension group, no relation was found between MVD and fracture both in men and women. In conclusion, MVD is an independent risk factor of vertebral fractures in old men. © 2017 The Authors. *JBMR Plus* is published by Wiley Periodicals, Inc. on behalf of the American Society for Bone and Mineral Research.

## Introduction

Bone loss and vascular abnormalities both occur insidiously and are initially asymptomatic processes that increase markedly with advancing age. As the number of elderly increases, so will the magnitude of the problem. Multiple factors including proteins, parathyroid hormone (PTH), phosphate, oxidized lipids, and vitamins D and K are implicated in both bone and vascular metabolism, illustrating the interaction of these two seemingly unrelated conditions.^1^ Some study has shown that diabetic microvascular complications contribute to risk of fracture.^2^, ^3^ For people who do not suffer from diabetes or hypertension with microvascular disease (MVD), the risk for fracture remains elusive. In addition, the association between MVD and fracture in large prospective study is limited. Evidence linking fragile fracture with microvascular complications in bone remains incompletely understood.

It is a well‐established principle that blood supply to the bone is a vital basis of bone growth and remodeling. Peripheral vascular resistance and the perfusion pressure gradient are two main factors controlling the rate of flow through the microvascular bed.^4^ Reduced blood flow has been linked low bone mass disorders. Two studies^5^, ^6^ have found that a special capillary subtype (CD31hi/Emcnhi vessels) and Notch signaling pathway are involved in the murine bone growth. The decline of CD31hi/Emcnhi vessels and the concomitant reduction of osteoprogenitor cells could potentially offer a compelling explanation for bone loss during aging.^5^ For technical difficulties, the precise overall structure of the skeletal vasculature in human has remained poorly understood. The human eye offers an excellent opportunity to visualize the microcirculation. Direct ophthalmoscopic examination provides a noninvasive means by which MVD can be assessed in vitro, and because the anatomy and physiology of retinal arterioles are similar to those of cerebral and coronary arterioles, retinal microcirculation abnormalities may reflect generalized microcirculatory pathology.^7^, ^8^ The spatial distribution of arteriole‐capillary connections determines regional differences in oxygenation and metabolic activity in bone.^5^ Calcaneal quantitative ultrasound (QUS) is a quick, simple, and inexpensive method free of ionizing radiation that appears to be effective in detecting bone loss.^9^ A great deal of literature^10^, ^11^, ^12^, ^13^, ^14^, ^15^, ^16^, ^17^, ^18^, ^19^, ^20^, ^21^ have shown that severe height loss (at least 2 cm over 3 to 7 years) is often a consequence of osteoporotic vertebral fractures, so we use height loss of at least 2 cm as a surrogate of vertebral fragile fractures.

Estrogen and age have a significant impact on bone mineral density (BMD)^22^ and vascularization.^23^ Therefore, we evaluate the different associations of microvascular abnormalities with bone loss/fracture among premenopausal women, postmenopausal women, men aged <65 years, and men aged ≥65 years. Because diabetes and hypertension can cause microangiopathy, we further categorized diabetic, hypertensive, and sex to explore the relations between the microvascular and vertebral fragile fractures.

## Materials and Methods

See the Supplemental Methods for more detail.

### Study cohort

**Figure 1:**
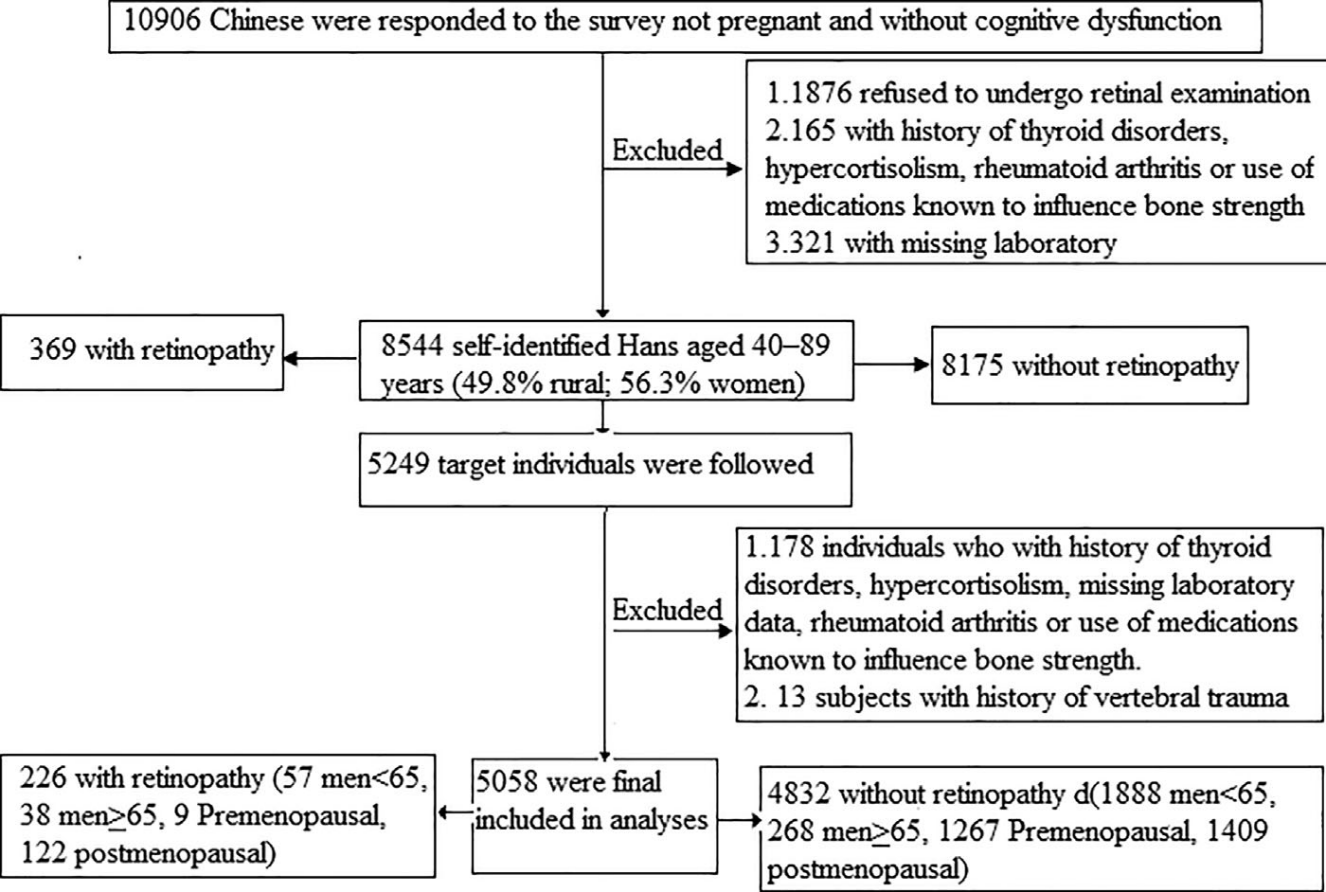
Study flow diagram.

This was a prospective observational cohorts study design is shown in reference 24 and supplemental material.^24^ A total of 10,906 Chinese responded to the survey who were not pregnant and had no cognitive dysfunction. Among them, 1876 subjects who refused to undergo retinal examination or were temporally outside, 165 with history of thyroid disorders, hypercortisolism, rheumatoid arthritis, or use of medications known to influence bone strength (ie, drugs for osteoporosis, thiazide diuretic, oral contraceptives, or glucocorticoids therapy), and 321 with missing laboratory data were excluded. The final analytic baseline sample included 8544 self‐identified Hans aged 40 to 89 years (49.8% rural; 56.3% women). Then baseline population was categorized into two groups, microvascular abnormalities and without microvascular abnormalities, according to the retinal examination result to follow. Three‐year follow‐up visits were conducted between August 2014 and November 2014. A total of 5058 participants were included in the final analysis, after excluding 178 individuals who had a history of thyroid disorders, hypercortisolism, rheumatoid arthritis, or use of medications known to influence bone strength (ie, drugs for osteoporosis, thiazide diuretics, oral contraceptives, or glucocorticoids therapy), and missing laboratory data and 13 subjects with a self‐reported history of vertebral trauma during the follow‐up (Fig. 1). Written informed consent was obtained from every participant, and the study has been approved by the Institutional Review Board of Fujian Provincial Hospital. All investigators received special training and were unaware of the aims of the study and were blinded to the characteristics of the subjects.

### Baseline assessment

BMD was assessed by scanning the left calcaneus with Sahara (Hologic, Inc., Waltham, MA, USA). All the parameters were measured twice by the same experienced operator, and the mean value was used for analysis. Results for calcaneal BMD were transformed to *T*‐scores (calculated as the difference between the actual measurement and the mean value of healthy sex‐matched adult controls, divided by their standard deviation), from the data provided by the densitometer manufacturer. According to the World Health Organization (WHO) criteria,^25^ bone status was categorized into three groups: normal BMD (*T*‐score ≥ −0.9), osteopenia (−2.4 ≤ *T*‐score ≤ −1.0), or osteoporosis (*T*‐score ≤ −2.5). Low BMD refers to T‐score ≤ −1.0.

Direct ophthalmoscopic examination from both eyes after 5 minutes of dark adaptation was conducted by two qualified retinal ophthalmologists independently. A research ophthalmologist undertook a quality assurance check of 10% of the diagnosis and no instances of discordance were identified. Microvascular abnormalities were defined as present if any of the following lesions were observed in any of the four fundus quadrants: microaneurysms, retinal hemorrhages (blot or flame shaped), soft exudates (cotton‐wool spots), hard exudates, macular edema, intraretinal microvascular abnormalities, venous beading, new vessels at the disc or elsewhere, vitreous hemorrhage, disc swelling, laser photocoagulation scars, arteriovenous nicking, or focal arteriolar narrowing.

Standing height was measured in light clothing without shoes with the use of an electronic stadiometer. Subjects placed both heels, buttocks, and their back against the stadiometer backboard, with the head positioned in the Frankfort horizontal plane. The participants were instructed to stretch to a fully erect position while keeping the feet flat on the floor. Excessive stretching was avoided. Besides the subjects’ heads being maintained in the Frankfort plane, the heads did not necessarily touch the backboard. The horizontal plate of the stadiometer was pressed firmly onto the heads, flattening the hair. Height was measured by certified nurses to the nearest 0.1 cm during normal respiration.^16^

A questionnaire including information on demographic characteristics, medical history, and lifestyle factors was administered. Anthropometric measurements were conducted. Body mass index (BMI) was calculated as weight (kg) divided by height (m) squared. Standard laboratory tests determined blood glucose, HbA1c, serum insulin, creatinine, and lipids. The index of homeostasis model assessment of insulin resistance (HOMA‐IR) was calculated as: fasting glucose (mmol/L) × fasting insulin (µU/mL)/22.5.^26^ The CKD‐EPI equation was used to estimate glomerular filtration rate (eGFR).^27^ Age is divided into four groups: ≤45, 46 to 55, 56 to 65, and >65 years.

Diabetes mellitus was defined as having fasting blood glucose (FBG) ≥ 7.0 mmol/L, porphobilinogen (PBG) ≥ 11.1 mmol/L, or history of treatment for diabetes. Insulin resistance (IR) was defined as HOMA‐IR higher than 2.50.^28^ Hypertension was defined as having systolic blood pressure (SBP) ≥ 140 mmHg, diastolic blood pressure (DBP) ≥ 90 mmHg, or history of treatment for hypertension. Dyslipidemia was defined as current treatment with cholesterol‐lowering medication or having one or more of the following: TC > 6.22 mmol/L, TG > 2.26 mmol/L, LDL > 4.4 mmol/L, and HDL < 1.04 mmol/L.^29^ Furthermore, general obesity was defined as BMI ≥ 27.5 kg/m^2^ and abdominal obesity as waist circumference (WC) ≥ 90 cm for men and ≥80 cm for women.^30^, ^31^ We defined abnormal eGFR as <60 mL/min/1.73 m^2^.^32^

### Collection of follow‐up data

Three‐year follow‐up visits measured height. Height loss of at least 2 cm over 3 years was an indication of vertebral fractures. Subjects self‐reported any history of trauma fracture(s), details of the way, site, and date. Methods of followed data collection were similar to the baseline. However, direct ophthalmoscopic examination and fasting insulin were not collected in the follow‐up visit. In addition, Calcaneal quantitative ultrasound was followed only in Wuyishan city.

### Statistical analysis

EpiData software (The EpiData Association, Odense, Denmark) was used to establish the database. All data were double entered in a database and then compared and corrected for errors. Continuous variables were shown by medians with interquartile ranges (IQR; the range between the 25th and 75th percentile) because of the non‐normal distribution, and categorical variables were expressed by counts and percentages. The differences among subjects in different groups were detected using the Kruskal‐Wallis test for continuous variables and chi‐square test for categorical variables. Constructing multiple logistic regression models determined the relationship between low BMD and microvascular abnormalities on baseline. First, the crude associations were evaluated (unadjusted) and then included adjustment for age (model 1). Subsequent models were built with inclusions of education, physical activity, smoking status, alcohol, coffee, milk, and bean products consumption (model 2), then further adjusted for diabetes, IR, hypertension, dyslipidemia, eGFR < 60 mL/min/1.73 m^2^, generalized and abdominal obesity, gastrointestinal disorders, respiratory diseases, and urological diseases (model 3). Additionally, binary logistic regression was used to analyze the association between vertebral fractures and microvascular abnormalities. The detailed process of adjusted confounding factors is given in Table 3. To further control the effects of diabetes mellitus and hypertension, data were split into diabetes mellitus and hypertension, in men and women, and the risk of vertebral fragile fractures with microvascular abnormalities were compared to without microvascular abnormalities by binary logistic regressions.

All data analyses were performed with SPSS 19.0 statistical software package (SPSS, Chicago, IL, USA). All *p* values were based on two‐sided tests, with statistical significance defined as *p* < 0.05.

## Results

At baseline, participants were randomly selected, with 56.3% being women and 43.7% being men, giving a sex ratio of 1.29:1, with a total median age of 53 years (IQR, 46 to 61 years). Prevalence of retinal microvascular abnormalities was 1.3%, 2.9%, 6.9%, and 9.6% in premenopausal women, men aged <65 years, postmenopausal women, and men aged ≥65 years, respectively, and low BMD prevalence was 10.7%, 28.3%, 40.6%, and 43.8% (Supplemental Table S1). The baseline measures, follow‐up measures, and percentage change in subjects with and without microvascular abnormalities are shown in Table 1. In the microvascular abnormalities group, the prevalence of vertebral fractures was 22.2%, 24.6%, 22.8%, and 39.5% among premenopausal women, men aged <65 years, postmenopausal women, and men aged ≥65 years, respectively (Supplemental Tables S2 and S3).

**Table 1:** Sex‐Specific Baseline and Followed‐up Characteristics

	Men			Women		
	Without retinopathy	With retinopathy	*p* Value	Without retinopathy	With retinopathy	*p* Value
Baseline						
Number	3577	158		4598	211	
Age (years)	53 (45 to 62)	63 (55 to 68)	0.001	51 (46 to 59)	62 (57 to 68)	0.001
High school or more	1285 (42.9)	278 (37.7)	0.161	921 (42.3)	696 (26.4)	0.001
Smokers	1709 (49.8)	106 (67.0)	0.001	28 (6.0)	5 (2.4)	0.013
Alcohol drinkers	1189 (33.2)	63 (39.9)	0.086	1205 (26.2)	40 (19.0)	0.001
Coffee drinkers	204 (5.7)	6 (3.8)	0.379	309 (6.7)	6 (2.8)	0.022
Milk drinkers	1831 (51.2)	84 (53.2)	0.684	2057 (44.7)	82 (44.5)	0.103
Bean products	2799 (78.2)	118 (74.7)	0.281	3681 (80.1)	155 (73.5)	0.023
Physical activity			0.001			0.001
Low	2399 (67.1)	123 (77.8)		3744 (81.4)	202 (95.7)	
Moderate	324 (9.1)	16 (10.1)		328 (7.1)	3 (1.4)	
High	854 (23.9)	19 (12.0)		526 (11.4)	6 (2.6)	
FBG (mmol/L)	5.5 (5.1 to 6.0)	6.2 (5.5 to 7.8)	0.001	5.4 (5.0 to 5.8)	6.2 (5.5 to 8.8)	0.001
PBG (mmol/L)	6.4 (5.1 to 8.2)	9.6 (6.8 to 15.4)	0.001	6.8 (5.7 to 8.3)	10.8 (7.7 to 11.8)	0.001
HbA1c (%)	5.7 (5.4 to 6.0)	6.2 (5.7 to 7.4)	0.001	5.7 (5.4 to 6.0)	6.3 (5.7 to 8.1)	0.001
FIN (μU/mL)	5.2 (3.4 to 7.7)	6.5 (4.3 to 9.8)	0.001	6.4 (4.6 to 8.8)	7.6 (5.3 to 11.3)	0.001
HOMA‐IR	1.3 (0.8 to 2.0)	2.1 (1.2 to 3.0)	0.001	1.5 (1.1 to 2.2)	2.2 (1.5 to 3.4)	0.001
SBP (mmHg)	134 (123 to 147)	146 (132 to 159)	0.001	127 (117 to 144)	145 (133 to 161)	0.001
DBP (mmHg)	78 (72 to 86)	85 (76 to 91)	0.001	76 (69 to 83)	78 (71 to 87)	0.001
HDL (mmol/L)	1.3 (1.1 to 1.5)	1.3 (1.1 to 1.5)	0.045	1.4 (1.2 to 1.6)	1.4 (1.2 to 1.6)	0.237
LDL (mmol/L)	2.9 (2.4 to 3.4)	2.8 (2.3 to 3.4)	0.209	3.0 (2.5 to 3.6)	3.1 (2.6 to 3.7)	0.018
TC (mmol/L)	5.0 (4.4 to 5.7)	5.1 (4.4 to 5.8)	0.810	5.2 (4.5 to 5.9)	5.5 (4.8 to 6.1)	0.001
TG (mmol/L)	1.4 (1.0 to 2.1)	1.6 (1.1 to 2.5)	0.006	1.3 (0.9 to 1.8)	1.6 (2.2 to 2.3)	0.001
BMI (kg/m^2^)	24.0 (21.9 to 26.2)	25.2 (23.2 to 27.5)	0.001	23.7 (21.8 to 25.9)	24.7 (22.6 to 27.3)	0.001
eGFR (mL/min/1.73 m^2^)	96.3 (86.1 to 104.0	84.7 (73.3 to 96.0)	0.001	96.9 (88.3 to 104.5	87.8 (74.9 to 94.4)	0.001
Diabetes	538 (15.0)	90 (57.0)	0.001	592 (12.9)	121 (57.3)	0.001
IR	506 (14.1)	63 (39.9)	0.001	830 (18.1)	91 (43.1)	0.001
Hypertension	1558 (43.6)	126 (79.7)	0.001	1717 (37.3)	165 (78.2)	0.001
Dyslipidemia	1466 (41.0)	74 (46.8)	0.160	1561 (33.9)	99 (46.9)	0.001
General obesity	498 (13.9)	40 (25.3)	0.001	641 (13.9)	50 (23.7)	0.001
Abdominal obesity	760 (21.2)	55 (34.8)	0.001	2048 (44.5)	146 (69.2)	0.001
eGFR <60 mL/min/1.73 m^2^	89 (2.5)	15 (9.5)	0.001	75 (1.6)	15 (7.1)	0.001
Gastrointestinal disorders	452 (12.6)	21 (13.3)	0.807	538 (15.0)	15 (7.1)	0.046
Respiratory diseases	112 (3.1)	7 (4.4)	0.350	83 (1.8)	3 (1.4)	0.999
Urological diseases	233 (6.5)	16 (10.1)	0.100	201 (4.4)	8 (3.8)	0.863
*T*‐score	–0.3 (–1.2 to 0.8)	–0.8 (–1.7 to 0.3)	0.001	0.1 (–1.0 to 1.4)	–0.6 (–1.6 to 0.5)	0.001
BMD status			0.001			0.001
Normal BMD	2475 (69.2)	88 (55.7)		3378 (73.5)	129 (61.1)	
Osteopenia	976 (27.3)	58 (36.7)		1032 (22.4)	63 (29.9)	
Osteoporosis	126 (3.5)	12 (7.6)		188 (4.1)	19 (9.0)	
Follow‐up						
Number	2156	95		2676	131	
Age (years)	54 (49 to 62)	63 (55 to 70)	0.001	53 (48 to 60)	65 (58 to 70)	0.001
Height loss at least 2 cm	345 (16.0)	28 (29.5)	0.002	519 (19.4)	32 (24.4)	0.176
Smokers	1165 (54.0)	42 (44.2)	0.073	10 (0.4)	4 (3.1)	0.003
Alcohol drinkers	768 (35.6)	29 (30.5)	0.086	81 (3.0)	4 (3.1)	0.001
Coffee drinkers	204 (5.7)	6 (3.8)	0.379	309 (6.7)	6 (3.8)	0.022
Milk drinkers	1831 (51.2)	84 (53.2)	0.684	2057 (44.7)	84 (53.2)	0.103
Physical activity			0.022			0.001
Low	1428 (66.2)	72 (75.8)		2234 (83.5)	126 (96.2)	
Moderate	216 (10.0)	12 (12.6)		201 (7.5)	3 (2.3)	
High	512 (23.7)	11 (11.6)		241 (9.0)	2 (1.5)	
FBG (mmol/L)	5.5 (5.2 to 6.0)	6.2 (5.5 to 7.8)	0.001	5.4 (5.1 to 5.9)	6.0 (5.3 to 8.1)	0.001
PBG (mmol/L)	7.0 (5.1 to 9.1)	9.6 (6.8 to 15.4)	0.001	7.1 (6.0 to 9.0)	11.1 (7.8 to 15.8)	0.001
HbA1c (%)	5.5 (5.2 to 5.8)	6.1 (5.5 to 7.2)	0.001	5.5 (5.3 to 5.8)	6.2 (5.6 to 7.4)	0.001
SBP (mmHg)	133 (122 to 144)	134 (144 to 154)	0.001	131 (118 to 145)	146 (136 to 157)	0.001
DBP (mmHg)	80 (73 to 87)	81 (74 to 95)	0.579	70 (77 to 84)	79 (71 to 85)	0.316
HDL (mmol/L)	1.3 (1.1 to 1.5)	1.2 (1.0 to 1.4)	0.027	1.4 (1.2 to 1.6)	1.3 (1.1 to 1.5)	0.001
LDL (mmol/L)	2.9 (2.4 to 3.3)	2.8 (2.3 to 3.3)	0.144	3.1 (2.6 to 3.7)	3.0 (2.5 to 3.6)	0.094
TC (mmol/L)	5.1 (4.5 to 5.8)	5.0 (4.3 to 5.6)	0.069	5.4 (4.7to 6.1)	5.3 (4.7 to 5.9)	0.162
TG (mmol/L)	1.5 (1.0 to 2.3)	1.7 (1.2 to 2.3)	0.166	1.4 (1.0 to 2.0.)	1.6 (1.2 to 2.4)	0.001
BMI (kg/m^2^)	24.0 (22.3 to 26.5)	26.8 (23.1 to 28.0)	0.001	24.0 (22.1 to 26.2)	25.3 (22.4 to 27.8)	0.001
eGFR (mL/min/1.73 m^2^)	95.3 (89.4 to 105.0	86.7 (80.6 to 92.5)	0.001	97.0 (91.6 to 101.6	88.3 (74.2 to 94.2)	0.001
Diabetes	337 (15.6)	30 (31.6)	0.001	437 (16.3)	36 (27.5)	0.002
Hypertension	1006 (46.7)	78 (82.1)	0.001	1140 (42.6)	116 (88.5)	0.001
Dyslipidemia	1094 (50.7)	57 (60.0)	0.093	1230 (46.0)	68 (51.9)	0.209
General obesity	498 (13.9)	40 (25.3)	0.001	641 (13.9)	40 (25.3)	0.001
Abdominal obesity	704 (32.7)	57 (60.0)	0.001	1605 (60.0)	107 (81.7)	0.467
eGFR <60 mL/min/1.73 m^2^	8 (0.4)	2 (2.1)	0.064	40 (1.5)	14 (10.7)	0.001
Gastrointestinal disorders	141 (6.5)	5 (5.3)	0.831	167 (6.2)	10 (7.6)	0.464
Respiratory diseases	40 (1.9)	3 (3.2)		18 (0.7)	1 (0.8)	0. 598
Urological diseases	137 (6.4)	13 (13.7)	0.010	94 (3.5)	6 (4.6)	0. 467
*T*‐score^a^	–1.2 (–1.9 to –0.4)	–1.5 (–2.5 to –0.4)	0.119	–0.9 (–1.7 to –0.1)	–1.6 (–2.2 to –0.8)	0.001
BMD status^a^			0.022			0.001
Normal BMD	826 (69.2)	30 (36.1)		1158 (52.1)	30 (25.2)	
Osteopenia	841 (27.3)	32 (38.6)		890 (40.0)	65 (54.6)	
Osteoporosis	232 (3.5)	21 (25.3)		176 (7.9)	24 (20.2)	
Follow‐up changing						
New smokers	159 (7.4)	7 (7.4)	0.999	6 (0.2)	2 (1.5)	0.050
New alcohol drinkers	184 (8.5)	11(11.6)	0.038	41 (1.5)	2 (1.5)	0.999
New diabetes	135 (6.3)	3 (3.2)	0.277	176 (6.6)	6 (4.6)	0.468
New hypertension	233 (10.8)	4 (4.2)	0.039	287 (10.7)	13(9.9)	0.885
New dyslipidemia	340 (15.8)	12 (12.6)	0. 473	273 (17.7)	17 (13.0)	0.195
New gastrointestinal disorders	94 (4.4)	4 (4.2)	0.999	108 (4.0)	5 (3.8)	0.999
New respiratory diseases	30 (1.4)	3 (3.2)	0.160	13 (0.5)	1 (0.8)	0.489
New urological diseases	98 (4.5)	8 (8.4)	0.084	75 (2.8)	5 (3.8)	0.421
General obesity changed	125 (5.8)	14(14.7)	0.002	162 (6.1)	14 (10.7)	0 .041
Abdominal obesity changed	386 (17.9)	21 (22.1)	0.174	687 (25.7)	23 (18.7)	0.077
eGFR <60 mL/min/1.73 m^2^ changed	36 (1.7)	7 (7.4)	0.002	48 (1.8)	12 (9.2)	0.001

HbA1c = glycated hemoglobin A1c; HOMA‐IR = homeostasis model assessment of insulin resistance; SBP = systolic blood pressure; DBP = diastolic blood triglycerides; HDL = high‐density lipoprotein cholesterol; LDL = low‐density lipoprotein cholesterol; TC = total cholesterol; TG = total triglycerides; BMI = body mass index; eGFR = estimated glomerular filtration rate; IR = insulin resistance.

Data are median (IQR) for continuous variables and count (%) for categorical variables. The difference among groups was examined by Kruskal‐Wallis test or chi‐square test where appropriate.

^a^ A total of 4325 subjects followed the measurement of calcaneal quantitative ultrasound (men without retinopathy, 1899; menwith retinopathy, 83; women without retinopathy, 2224; women with retinopathy, 1119).

### Retinal microvascular abnormalities and low BMD risk

In men aged ≥65 years, compared with participants without retinal microvascular abnormalities, those with retinal microvascular abnormalities had significantly higher risk of low BMD even after adjustment for different confounders; the fully adjusted odds ratio (OR) was 2.506 (95% confidence interval [CI] 1.454–4.321; *p* = 0.001). However, no significant associations were found between retinal microvascular abnormalities and low BMD risk in men aged <65 years and premenopausal or postmenopausal women (all *p* > 0.05) (Table 2).

**Table 2:** Retinopathy and Low BMD Risk (OR [95% CI])

	Retinopathy		
	None	Present	*p* Value
Men			
<65 years (*n* = 2998)			
No. of cases	2911	87	
Unadjusted	1.00	1.491 (0.957, 2.323)	0.077
Model 1	1.00	1.336 (0.855, 2.089)	0.203
Model 2	1.00	1.272 (0.811, 1.995)	0.295
Model 3	1.00	1.421 (0.890, 2.268)	0.141
≥65 years (*n* = 737)			
No. of cases	666	71	
Unadjusted	1.00	1.539 (0.942, 2.515)	0.085
Model 1	1.00	1.620 (0.987, 2.660)	0.056
Model 2	1.00	1.899 (1.142, 3.158)	**0.013**
Model 3	1.00	2.506 (1.454, 4.321)	**0.001**
Women			
Premenopausal (*n* = 2176)			
No. of cases	2147	29	
Unadjusted	1.00	1.753 (0.663, 4.641)	0.258
Model 1	1.00	0.916 (0.325, 2.581)	0.868
Model 2	1.00	0.918 (0.322, 2.621)	0.873
Model 3	1.00	1.275 (0.413, 3.939)	0.673
Postmenopausal (*n* = 2633)			
No. of cases	2451	182	
Unadjusted	1.00	1.079 (0.795, 1.463)	0.627
Model 1	1.00	0.764 (0.556, 1.051)	0.098
Model 2	1.00	0.764 (0.555, 1.053)	0.100
Model 3	1.00	0.861 (0.618, 1.199)	0.376

Data are ORs (95% CI). Bold indicates statistically significant (*p* < 0.05). Each model included the following covariates for adjustment: model 1: age; model 2: model 1 + education, physical activity, smoking status, alcohol, coffee, milk, and bean products consumption; model 3: model 2 + diabetes, insulin resistance, hypertension, dyslipidemia, eGFR <60 mL/min/1.73 m^2^, generalized and abdominal obesity, gastrointestinal disorders, respiratory diseases, and urological diseases.

### Retinal microvascular abnormalities and vertebral fractures risk

As shown in Table 3, retinal microvascular abnormalities were associated with the risk of vertebral fractures in men aged ≥65 years, OR was 2.475 (95% CI 1.085–5.646; 0.031). However, no significant associations were found between retinal microvascular abnormalities and vertebral fractures in premenopausal and postmenopausal women and men aged <65 years (all *p* > 0.05). When stratified by diabetes, in the without‐diabetes group, men with retinal microvascular abnormalities had higher risk for vertebral fractures than without retinopathy (OR = 2.194; 95% CI = 1.097–4.389; *p* = 0.026) after adjusted confounding factor. However, the difference was not found in women. In the diabetes group, both in men and women, the risk for vertebral fractures between those with retinal microvascular abnormalities and those without were not significantly different. Stratified by hypertension, hypertensive men with retinopathy had higher risk for vertebral fractures than those without (OR = 2.034; 95% CI 1.163–3.559; *p* = 0.013), but the difference was not found among women. In the without‐hypertension group, no associations were found between MVD and fracture both in men and women (Table 4).

**Table 3:** Retinopathy and Vertebral Risk (ORs [95%CI])

	Retinopathy		
	None	Present	*p* Value
Men			
<65 years (*n* = 1945)			
No. of cases	1888	57	
Unadjusted	1.00	1.690 (0.899, 3.177)	0.104
Model 1	1.00	1.407 (0.743, 2.663)	0.294
Model 2	1.00	1.499 (0.786, 2.861)	0.219
Model 3	1.00	1.470 (0.752, 2.874)	0.260
Model 4	1.00	1.403 (0.713, 2.760)	0.327
≥65 (*n* = 306)			
No. of cases	268	38	
Unadjusted	1.00	2.079 (1.024, 4.222)	**0.043**
Model 1	1.00	1.981 (0.966, 4.063)	0.062
Model 2	1.00	2.277 (1.650, 4.867)	**0.034**
Model 3	1.00	2.514 (1.117, 5.656)	**0.026**
Model 4	1.00	2.475 (1.085, 5.646)	**0.031**
Women			
Premenopausal (*n* = 1276)			
No. of cases	1267	9	
Unadjusted	1.00	1.552 (0.320, 7.525)	0.585
Model 1	1.00	1.630 (0.335, 7.921)	0.545
Model 2	1.00	1.358 (0.271, 6.798)	0.709
Model 3	1.00	1.338 (0.248, 7.231)	0.735
Model 4	1.00	1.387 (0.249, 7.728)	0.709
Postmenopausal (*n* = 1531)			
No. of cases	1409	122	
Unadjusted	1.00	1.101 (0.716, 1.693)	0.662
Model 1	1.00	1.008 (0.650, 1.563)	0.972
Model 2	1.00	1.017 (0.654, 1.582)	0.940
Model 3	1.00	0.995 (0.631, 1.571)	0.983
Model 4	1.00	0.972 (0.613, 1.541)	0.904

Data are ORs (95%CI). Bold indicates statistically significant (*p* < 0.05). Each model included the following covariates for adjustment: model 1: grouped age, baseline BMD; model 2: model 1 + education, physical activity, smoking status, alcohol, coffee, milk, and bean products consumption; model 3: model 2 + diabetes, insulin resistance, hypertension, dyslipidemia, eGFR <60 mL/min/1.73 m^2^, generalized and abdominal obesity; model 4: model 3 + the new occurrence of smoking status, alcohol, diabetes, hypertension, dyslipidemia, and the change of generalized and abdominal obesity, eGFR <60 mL/min/1.73 m^2^.

**Table 4:** Prevalence and ORs of Vertebral Fracture Among Different Sex Retinopathy Stratified by Diabetes and Hypertension

	Men retinopathy		Women retinopathy	
	Without	With	Without	With
No. without diabetes	1750	43	2225	59
Prevalence %	15.5	32.6	19.1	25.4
OR (95% CI)^a^	1.0 (ref)	2.194 (1.097, 4.389)	1.0 (ref)	1.010 (0.545, 1.874)
*p*		**0.026**		0.974
No. with diabetes	406	52	451	72
Prevalence %	18.0	26.9	21.1	23.6
OR (95% CI)^a^	1.0 (ref)	1.358 (0.651, 2.832)	1.0 (ref)	0.978 (0.501, 1.908)
*p*		0.414		0.978
No. without hypertension	1150	17	1536	15
Prevalence %	15.3	23.5	18.2	33.3
OR (95% CI)^a^	1.0 (ref)	1.349 (0.400, 4.551)	1.0 (ref)	1.625 (0.509, 5.189)
*p*		0.630		0.412
No. with hypertension	1006	78	1140	116
Prevalence %	16.8	30.8	21.0	23.3
OR (95% CI)^a^	1.0 (ref)	2.034 (1.163, 3.559)	1.0 (ref)	0.964 (0.593, 1.568)
*p*		**0.013**		0.883

^a^ All ORs use the without‐retinopathy cohort as the reference cohort (ref).

ORs were adjusted for age, baseline BMD, education, physical activity, smoking status, alcohol, coffee, milk, and bean products consumption, dyslipidemia, eGFR <60 mL/min/1.73 m^2^, generalized and abdominal obesity, the new occurrence of smoking status, alcohol, diabetes, hypertension, dyslipidemia, and the change of generalized and abdominal obesity, eGFR <60 mL/min/1.73 m^2^. In addition, adjustment for diabetes when stratified by hypertension and adjustment for hypertension when stratified by diabetes.

Bold indicates statistically significant (*p* < 0.05).

## Discussion

To our knowledge, the present study is the first attempt to explore the association between the presence of retinal microvascular abnormalities and BMD/vertebral fractures stratified by age, sex, menopausal status, diabetes, and hypertension in a large prospective study. In the baseline study, our team found that participants with retinal microvascular abnormalities have a higher risk of low BMD compared with participants without microvascular abnormalities after adjustment for confounders among men aged ≥65 years. After participants’ height were followed for an average of 2.93 years, results showed that risk for vertebral fractures (height loss of at least 2 cm) was 2.475 times higher compared with the participants without retinal microvascular abnormalities among men aged ≥65 years. In addition, when stratified by diabetes, in no diabetic group, men with microvascular abnormalities had higher risk for vertebral fracture compared with participants without retinopathy. Stratified by hypertension, hypertensive men with microvascular abnormalities had a higher risk for vertebral fracture compared with participants without hypertension.

The current result showed that retinal microvascular abnormalities were associated with an increased risk of low BMD and vertebral fragile fracture(s). These findings are consistent with the notion that there is an association between vascular disease and bone. In this prospective study, not only baseline results showed that men aged ≥65 years with MVD had higher risk of low BMD compared with participants without microvascular abnormalities, but also after 3‐year follow‐up visits, the men aged ≥65 years with MVD had a higher risk of fracture after adjustment for age, BMD, and other serial factors. This result indicated that MVD can lead to low BMD and fracture(s), although various common risk factors between BMD and MVD may lead to interaction of these two. MVD is an independent risk factor of vertebral fractures in old men. Shanbhogue and colleagues’ analysis of adult type 1 diabetes patients with and without diabetic MVD compared with healthy control subjects, respectively, show that the presence of MVD was associated with deficits in cortical and trabecular bone volumetric BMD and microarchitecture that could partly explain the excess skeletal fragility.^33^ Ivers and colleagues in a prospective study showed increased risk of fracture associated with diabetic retinopathy.^3^ However, in our study, we observed no diabetic men with retinopathy had an increased risk of vertebral fracture after adjusted age, BMD, and other confounding factors. The difference was not found in the diabetic subjects group. This heterogeneity may be because skeletal fragility often accompanies diabetes, but diabetes affects bone via many pathophysiological mechanisms, such as impairments in glucose metabolism and toxic effects of glucose oxidative derivatives, and via impairments in bone microvascular function and muscle endocrine function.^34^ So we did not find a difference of risk for vertebral fracture in the diabetic subjects’ group. Stratified by hypertension, only hypertensive men with microvascular abnormalities had higher risk for vertebral fracture compared with participants without retinopathy after adjustment for age, BMD, diabetes, and other confounders. The difference was not found in subjects without hypertension.

Current research confirms the study by Kusumbe and colleagues, which reported that the reduction of CD31hi/Emcnhi vessels (a special subtype of capillaries that was almost unique to bone) and the concurrent decline of osteoprogenitor cells could potentially offer a compelling explanation for bone loss in mice during aging.^5^ CD31hi/Emcnhi endothelial cells mediate vasculature growth and provide niche signals for perivascular osteoprogenitors. Impaired vascular growth and bone loss were found in the genetically modified mice with disrupted Notch signaling in endothelial cells.^6^ Researchers have shown that improved angiogenesis in bone benefits bone formation. For example, platelet‐derived growth factor‐BB, exogenous or released by preosteoclasts, induces formation of the CD31hi/Emcnhi vessel subtype to promote the coupling of angiogenesis with bone formation;^35^ exogenous factors DJ‐1 induce osteogenesis and angiogenesis, both of which have properties that are essential for bone regeneration.^36^ Our findings suggest that retinal microangiopathy may, as an index of the disturbed bone vascularity (declined CD31hi/Emcnhi vessels and impaired Notch signaling), be a mechanism leading to bone loss by diminished bone formation and, perhaps, excessive bone resorption, which affect BMD and lead to an increased risk for fractures in men aged ≥65 years. Moreover, administration of antiangiogenic drugs are associated with adverse effects in vascular homeostasis and endocrine organs.^37^ If our findings are confirmed in other samples, regular ophthalmoscopy and calcaneus quantitative ultrasound are needed among patients with antiangiogenic therapies.

However, the association between retinal microvascular abnormalities and low BMD risk and vertebral fractures has not been found in men aged <65 years and premenopausal and postmenopausal women. It is well known that women after menopause have accelerated bone loss, suggesting that estrogen deficiency plays a major role in this loss, which may cover the effect of microvascular abnormalities on low BMD. This may partly explain why we fail to detect the association in postmenopausal women.

A strength of this work is that it was a prospective cohorts study, which included a large sample size of both men and women drawn from the general population rather than a specialized sample. The standardized identification of retinal microvascular abnormalities, detailed information collected on a range of risk factors, and potential confounders were collected. Our study examined the different associations of calcaneal BMD and fracture risk with microvascular abnormalities stratified by sex, age, menstrual status, diabetes, and hypertension. Limitations should also be stated. It was indirect to use height loss as a surrogate of vertebral fractures instead of imaging examination, since height loss may be due to weakening of the muscle groups, postural changes, disc degeneration, joint space narrowing, and spinal deformities. Also, the current study used calcaneal ultrasound *T*‐scores, not by conventional dual‐energy X‐ray absorptiometry. Calcaneal quantitative ultrasound at commonly used cutoff thresholds do not definitively exclude or confirm DXA‐determined osteoporosis.^38^ Additionally, the inclusion of only persons who are Hans threatens the study's generalizability because patterns of bone density are known to vary by ethnicity.^39^ Furthermore, many other variables potentially related to the risk of having low BMD, such as level of vitamin D, sex hormones, PTH, and other unknown or less clearly understood risk factors (for instance, genetic and inflammatory markers), may have played a role or modified the associations that we did not collect or assess because of the financial constraints. In addition, BMD was measured from a single bone site. However, the measurement of different sites of BMD in a multicenter epidemiologic study like this has not proven to be easy or practical, especially in remote mountainous regions.

In summary, our results provide evidence that retinal microvascular abnormalities are an independent risk factor of osteoporosis fracture in elderly men.

## Disclosures

All authors state that they have no conflicts of interest.

## Supporting information

Additional supporting information may be found in the online version of this article at the publisher's web‐site.

Supporting Data S1.
