# Supplementary material for: Is Retinal Microvascular Abnormalities an Independent Risk Factor of Vertebral Fractures? A Prospective Study From a Chinese Population
Source: JBMR Plus. 2017 Oct 10;1(2):107–15. doi: 10.1002/jbm4.10017 (PMC6124164; doi:10.1002/jbm4.10017)
Supplement: Supplementary file 1 — Supporting Data S1. [file JBM4-1-107-s001.doc]

**SUPPLEMENTAL** **MATERIAL**

Supplemental materials include five sections

1. Supplemental methods
2. Supplemental results
3. RACRION group
4. References
5. Supplemental Tables

**SUPPLEMENTAL METHODS**

**Study cohort**

The subjects registered in our study were permanent residents in Fujian province southeast of China from June 2011 to January 2012. A total of seven counties in Ningde city, six counties in Wuyishan city and eight communities (four from each city) were randomly selected using a clustered sampling technique with probabilities proportionate to the size of the population in each cluster. We contacted the leaders of the target villages and explained the importance of the study. Local media such as television and radio was also used to publicize the study. Depending on the village selected, individuals were eligible if they were 40 years of age or older and were resident at least ten years in the target villages without cognitive dysfunction and were not pregnant. A letter was sent to each potential subject’s house inviting him/her to participate in the study. At least a week before, a visit to the potential subject’s home was made by local organizers (usually the leaders of the village and/or village doctors) and the nature of the study was explained in detail by recruitment staff (one clinician and two assistants). Each participant was invited for a funduscopy, measurement of brachial-ankle pulse wave velocity (baPWV) and ankle brachial index (ABI), a standardized questionnaire, physical examinations, and blood sample collections. All the examinations were provided for free and a senior ophthalmologist explained the results of the evaluations to the participants. In order to overcome language barriers and trust issues, all interviewers and coordinators were employed from local hospital or medical schools. Conversations conducted by the staff were carried out in the local dialect. Participation in the study was voluntary. Finally, a total of 10906 Chinese responded to the survey from Ningde (4442) and Wuyishan（6464）. Among them, 360 are minority people in Ningde and they all refused to undergo retinal examination. Some Hans also declined. The base population of the current analysis therefore included 9030 self-identified Han participants who join in the microangiopathy study (response rate 82.80%). We excluded 165 participants with history of thyroid disorders, [hypercortisolism](http://cn.bing.com/dict/clientsearch?mkt=zh-CN&setLang=zh&form=BDVEHC&q=皮质醇增多症), rheumatoid arthritis or use of medications known to influence bone strength (i.e., drugs for osteoporosis, thiazide diuretics, oral contraceptives, or glucocorticoids therapy) and 321 with missing laboratory data. The final analytic sample included 8544 members aged 40–89 years (49.8% rural; 56.3% women). Then baseline population was categorized into two groups, microvascular abnormalities and without microvascular abnormalities, to follow up three years. The target participants we followed are in total of 5249 (follow-up rate 61.4%). 5058 participants were included into final analyses after excluded 178 individuals who with history of thyroid disorders, [hypercortisolism](http://cn.bing.com/dict/clientsearch?mkt=zh-CN&setLang=zh&form=BDVEHC&q=皮质醇增多症), rheumatoid arthritis, or use of medications known to influence bone strength (i.e., drugs for osteoporosis, thiazide diuretics, oral contraceptives, or glucocorticoids therapy) or missing laboratory data and 13 subjects self-report with history of vertebral trauma. All investigators received special training before the investigations were unaware of the aims of the study and were blinded to the characteristics of the subjects. Written informed consent was obtained from every participant before data collection, those who can’t read or write were asked to stamp with right forefingers and the study has been approved by the Institutional Review Board of Fujian Provincial Hospital.

**Data collection**

Data collection was conducted in examination centers at local health stations or community clinics in the participants’ residential area.

**Bone mineral density (BMD) measurements and definition of bone status**

BMD was assessed by scanning the left calcaneus (the right calcaneus was scanned only if measurement on the left was impossible, e.g. due to recent fracture or skin injury) with Sahara (Hologic, Inc., Waltham, Massachusetts, USA). Standardization and calibration of each QUS instruments were performed prior to the first measurement of each survey day. All the ultrasound parameters were measured twice by the same experienced operator on the same machine following the manufacturers’ recommendations and the study protocol, and the mean value was used for analysis. Results for calcaneal BMD were transformed to T-scores (calculated as the difference between the actual measurement and the mean value of healthy gender-matched adult controls, divided by their standard deviation), from the data provided by the densitometer manufacturer.

According to the World Health Organization (WHO) criteria, bone status were categorized into three groups: normal BMD (T-score>﹣0.9), osteopenia (﹣2.4<T-score<﹣1.0), or osteoporosis (T-score<﹣2.5). Low BMD refers to T-score<﹣1.0.

**Funduscopy and definition of microvascular disease**

Direct ophthalmoscopic examination was performed independently by two qualified retinal ophthalmologists. They conducted the detailed fundus examination of the retinas from both eyes after five minutes of dark adaptation following standardized protocols. A research ophthalmologist undertook a quality assurance check of 10% of the diagnosis, following audit specifications of the national screening guidelines. As a result of this process, no instances of discordance in diagnosis were identified.

Retinopathy was defined as present if any of the following lesions were examined definite in any of the four fundus quadrants: microaneurysms, retinal hemorrhages (blot or flame shaped), soft exudates (cotton-wool spots), hard exudates, macular edema, intraretinal microvascular abnormalities, venous beading, new vessels at the disc or elsewhere, vitreous hemorrhage, disc swelling, laser photocoagulation scars, arteriovenous nicking, or focal arteriolar narrowing.

**Measurement of height and definition of vertebral fractures**

Height was measured respectively to the nearest 0.1 cm by certified nurses with subjects without shoes, with the heels, buttocks, and back to the stadiometer backboard. The patients were instructed to stretch to a fully erect position while keeping the feet flat on the floor. Excess stretching was avoided. Besides the subjects’ heads were maintained in the Frankfort plane and heads did not necessarily touch the backboard. The horizontal plate of the stadiometer was pressed firmly onto the heads, flattening the hair. A lot of literature has shown that severe height loss (at least two centimeters over three–seven years) is often a consequence of osteoporotic vertebral fractures. We determined prospective height loss (difference between the current height and baseline documented height measurement) at least two centimeters as a detection of vertebral fractures.

**Questionnaire**

Educational levels were categorized into five groups: illiteracy/half-illiteracy, primary school, middle school, high school or equivalent, college and above. Illiteracy was defined as the disability to read any Chinese word. Half-illiteracy was present if the person could understand some of Chinese words, but could not get any useful information from the reading. Physical activity was measured as metabolic equivalent task [MET]-hours per week (MET-h/wk) and then categorized into three groups according to the Global Physical Activity Questionnaire (GPAQ) Analysis Guide: (i) High: Vigorous-intensity activity on at least three days achieving a minimum of at least 1,500 MET-minutes/week OR seven or more days of any combination of walking, moderate- or vigorous- intensity activities achieving a minimum of at least 3,000 MET-minutes per week ; (ii) Moderate: A person not meeting the criteria for the “high” category, but meeting any of the following criteria: three or more days of vigorous-intensity activity of at least 20 minutes per day, OR five or more days of moderate-intensity activity or walking of at least 30 minutes per day, OR five or more days of any combination of walking, moderate- or vigorous intensity activities achieving a minimum of at least 600 MET-minutes per week; and (iii) Low: A person not meeting any of the above mentioned criteria falls in this category. Smokers included current and ex-smokers, current smokers were those who smoked at least one cigarette per day or seven cigarettes per week lasting for at least half a year; ex-smokers were those who had regularly smoked in the past, but had quit for at least half a year. Non-smokers were those who never or only occasionally smoked. Alcohol drinkers were those who drank six g or more alcohol per day on average for at least half a year and non-drinkers were those who never or only occasionally drank. Coffee drinkers were those who drank one or more cup of coffee per day on average for at least half a year. Milk drinkers and bean products intake were those who self-reported milk or bean products (e.g. tofu) consumption during the past year. Gastrointestinal disorders, respiratory diseases, and urological diseases were present according to self-reported physician diagnosis. New smokers, new alcohol drinkers, new gastrointestinal disorders, new respiratory diseases, and new urological diseases were determined changed in the period of follow-up.

**Physical examinations**

After subjects had rested in a sitting position for more than five min, blood pressure was measured using a standard OMRON auto-electronic-sphygmomanometer (OMRON Model HEM-7117, Omron Company, Dalian, China) early in the morning prior to drawing blood samples. Systolic and diastolic blood pressures（SBP and DBP） were both measured three times consecutively at one-min intervals and the mean of the three readings was used for analysis. Weight, height, waist and hip circumference were measured respectively to the nearest 0.1 kg and 0.1 cm by certified nurses with subjects lightly clothed and without shoes. Body mass index (BMI) was calculated as weight in kilograms divided by height in meters squared (kg/㎡). Waist circumference (WC) was measured at the midpoint between the costal margin.

**Laboratory tests**

Subjects were requested to fast for at least ten hours prior to blood drawing. From the fasting venous blood specimens, standard laboratory tests determined fasting blood glucose（FBG）, fasting serum insulin (FIN), serum creatinine (SCr) , and lipid profile, including total cholesterol（TC）, total triglycerides（TG）, low density lipoprotein cholesterol (LDL-C) , and high density lipoprotein cholesterol (HDL-C) . Participants with no history of diabetes were administered a standard 75-g oral glucose tolerance test (OGTT). For Chinese medical ethics, DM subjects received 100 g of steamed bread for surrogate. 120 min later, five milliliters blood with anticoagulant was collected for measurement of postprandial blood glucose (PBG). Blood glucose levels were determined by the glucose oxidase method (Sclavo, Siena, Italy) and HbA1c was measured by an automated ion-exchange chromatographic method (Bio-Rad, Hercules, CA). FIN, SCr, TC, TG, LDL, and HDL was measured using chemiluminescence methods on the autoanalyzer (Modular E170; Roche). All samples were analyzed in the same central laboratory. The quality control in the laboratory was certified and monitored monthly by Ministry of Health, China. The index of homeostasis model assessment of insulin resistance (HOMA-IR) was calculated as: fasting glucose (mmol/l) × fasting insulin (μU/ml)/22.5. The CKD-EPI equation was used to estimate glomerular filtration rate expressed in milliliters per minute per 1.73 m²: estimated glomerular filtration rate (eGFR).

**Diagnostic criteria of risk factors**

Diabetes mellitus was defined as FBG>7.0 mmol/L or PBG>11.1 mmol/L, or self-reported history of diabetes or use of antidiabetic medications. Insulin resistance (IR) was defined as HOMA-IR higher than 2.50. Hypertension was defined as SBP>140 mmHg and/or DBP>90 mmHg, or self-reported history or treatment for hypertension. Dyslipidemia was defined as self-reported current treatment with cholesterol-lowering medication or having one or more of the following : TC>6.22mmol/L (240mg/dL), TG>2.26mmol/L (200mg/dL), LDL>4.14mmol/L (160mg/dL), HDL<1.04mmol/L (40mg/dL). General obesity was defined as BMI>27.5 kg/㎡ according to WHO suggestions for Chinese and abdominal obesity: WC>90 cm for men and >80 cm for women. We defined abnormal glomerular filtration rate as eGFR<60ml/min/1.73m². New diabetes, new hypertension, new dyslipidemia, change of obesity changed, change of abdominal obesity, and change of eGFR<60ml/min/1.73m² are confirmed during followed up.

**Statistical analysis**

EpiData software (The EpiData Association, Odense, Denmark) was used to establish the database. All data were double entered in a database and then compared and corrected for errors. Because sex, age, and menstrual status have significant impact on BMD and, to better identify the relationships between BMD and angiopathy, we conducted the analysis of men stratified by age and women stratified by menopausal status, i.e. men<65, men>65, premenopausal women, and postmenopausal women. Continuous variables were shown by medians with inter-quartile ranges (IQR, the range between the 25th and 75th percentile) due to the non-normal distribution and categorical variables were expressed by counts and percentages. The differences among subjects in different groups were detected using the Kruskal-Wallis test for continuous variables and Chi-squared test for categorical variables.

Constructing multiple logistic regression models determined the relationship between low BMD and microvascular abnormalities on baseline.. All analyses included adjustment for age (Model 1). Subsequent models were built with staged inclusions of education, physical activity, smoking status, alcohol, coffee, milk, and bean products consumption (Model 2), then further adjusted for diabetes, IR, hypertension, dyslipidemia, eGFR<60ml/min/1.73 ㎡, generalized and abdominal obesity, gastrointestinal disorders, respiratory diseases, and urological diseases (Model 3). Additionally, binary logistic regression was used to analyze the vertebral fractures and microvascular lesions. To further control the effects of diabetes mellitus and hypertension, data were spit into diabetes mellitus and hypertension, in men and women, the risk of vertebral fragile fractures with microvascular abnormalities were compared to without microvascular abnormalities by binary logistic regressions. The detail process of adjusted confounding factors referred to Table 3.

All data analyses were performed with SPSS 19.0 statistical software package (SPSS, Chicago, IL, USA). All P-values were based on two-sided tests, with statistical significance defined as P < 0.050

**SUPPLMENTAL** **RESULTS**

**Baseline Characteristics for subjects with and without retinal microvascular abnormalities**

Compared with individuals without retinal microvascular abnormalities, people with microangiopathy were more likely to be older, postmenopausal women, smokers, without bean products consumption, low physical activity, lower level of HDL, eGFR, and T-score, and higher level of FBG, PBG, HbA1c, FIN, HOMA-IR, SBP, DBP, TC, TG, and BMI, and higher prevalence of diabetes, IR, hypertension, dyslipidemia, general obesity, abdominal obesity, abnormal eGFR, and low BMD, but they were less likely to be coffee drinkers (Supplemental Table 1).

**Baseline and followed Characteristics for subjects followed with and without retinopathy**

Our team compared subjects with retinal microvascular abnormalities to without among men age<65, men age>65, premenopausal women and postmenopausal women. Four groups with microvascular abnormalities were higher prevalence of diabetes, IR, hypertension, abdominal obesity. The detail other confounding factors can see from (Supplemental Table 2-1, Table 2-2).

**REACTION STUDY GROUP**

**Steering Committee:** Guang Ning (Principle Investigator), National Clinical Research Center for Metabolic Diseases, Rui Jin Hospital, Shanghai Jiao Tong University School of Medicine, Shanghai, China; Yiming Mu, People’s Liberation Army General Hospital, Beijing, China; Jiajun Zhao, Shandong Provincial Hospital, Jinan, China; Weiqing Wang, National Clinical Research Center for Metabolic Diseases, Rui Jin Hospital, Shanghai Jiao Tong University School of Medicine, Shanghai, China; Chao Liu, Jiangsu Province Hospital on Integration of Chinese and Western Medicine, Nanjing, China; Yufang Bi, National Clinical Research Center for Metabolic Diseases, Rui Jin Hospital, Shanghai Jiao Tong University School of Medicine, Shanghai, China; Donghui Li, Department of Gastrointestinal Medical Oncology, the University of Texas MD Anderson Cancer Center, Houston, Texas, USA; Shenghan Lai, Johns Hopkins University School of Medicine, Baltimore, Maryland, USA; Zachary T. Bloomgarden, Mount Sinai School of Medicine, New York, USA.

**Working Group:** Weiqing Wang, Yufang Bi, Jieli Lu, National Clinical Research Center for Metabolic Diseases, Rui Jin Hospital, Shanghai Jiao Tong University School of Medicine, Shanghai, China; Yiming Mu, People’s Liberation Army General Hospital, Beijing, China; Jiajun Zhao, Shandong Provincial Hospital, Jinan, China; Chao Liu, Jiangsu Province Hospital on Integration of Chinese and Western Medicine, Nanjing, China; Lulu Chen, Wuhan Xiehe Hospital, Huazhong University of Science and Technology School of Medicine, Wuhan, China; Lixin Shi, Affiliated Hospital of Guiyang Medical College, Guiyang, China; Qiang Li, The Second Affiliated Hospital of Haerbin Medical University, Haerbin, China; Tao Yang, The First Affiliated Hospital with Nanjing Medical University, Jiangsu Province Hospital, Nanjing, China; Li Yan, Sun Yat-sen Memorial Hospital, Sun Yat-sen University, Guangzhou, China; Qin Wan, The Affiliated Hospital of Luzhou Medical College, Luzhou, China; Shengli Wu, Xinjiang Kelamayi Peoples Hospital, Kelamayi, China; Guixia Wang, The First Hospital of Jilin University, Changchun, China; Zuojie Luo, The First Affiliated Hospital of Guangxi Medical University, Nanning, China; Xulei Tang, The First Hospital of Lanzhou University, Lanzhou, China; Gang Chen, Fujian Provincial Hospital, Fujian Medical University, Fuzhou, China; Yanan Huo, Jiangxi People's Hospital, Nanchang, China; Zhengnan Gao, Dalian Municipal Central Hospital, Dalian, China; Qing Su, Xinhua Hospital, Shanghai Jiao Tong University School of Medicine, Shanghai, China; Zhen Ye, Zhejiang Center for Disease Control, China; Youmin Wang, The First Affiliated Hospital of Anhui Medical University, Hefei, China; Guijun Qin, The First Affiliated Hospital of Zhengzhou University, Zhengzhou, China; Huacong Deng, The First Affiliated Hospital of Chongqing Medical University, Chongqing, China; Xuefeng Yu, Wuhan Tongji Hospital, Huazhong University of Science and Technology School of Medicine, Wuhan, China; Feixia Shen, The First Affiliated Hospital of Wenzhou Medical University, The First Provincial Wenzhou Hospital of Zhejiang, Wenzhou, China; Li Chen, Qilu Hospital, University of Shandong School of Medicine, Jinan, China.

**SUPPLEMENTAL REFERENCES**

1. Kanis JA, Melton LJ, 3rd, Christiansen C, Johnston CC, Khaltaev N. The diagnosis of osteoporosis. Journal of bone and mineral research : the official journal of the American Society for Bone and Mineral Research. Aug 1994;9(8):1137-41. Epub 1994/08/01.

2. Siminoski K, Warshawski RS, Jen H, Lee K. The accuracy of historical height loss for the detection of vertebral fractures in postmenopausal women. Osteoporosis international : a journal established as result of cooperation between the European Foundation for Osteoporosis and the National Osteoporosis Foundation of the USA. Feb 2006;17(2):290-6. Epub 2005/09/07.

3. Cosman F, de Beur SJ, LeBoff MS, Lewiecki EM, Tanner B, Randall S, et al. Erratum to: Clinician's guide to prevention and treatment of osteoporosis. Osteoporosis international : a journal established as result of cooperation between the European Foundation for Osteoporosis and the National Osteoporosis Foundation of the USA. Jul 2015;26(7):2045-7. Epub 2015/05/20.

4. Masunari N, Fujiwara S, Kasagi F, Takahashi I, Yamada M, Nakamura T. Height loss starting in middle age predicts increased mortality in the elderly. Journal of bone and mineral research : the official journal of the American Society for Bone and Mineral Research. Jan 2012;27(1):138-45. Epub 2011/09/21.

5. Xu W, Perera S, Medich D, Fiorito G, Wagner J, Berger LK, et al. Height loss, vertebral fractures, and the misclassification of osteoporosis. Bone. Feb 2011;48(2):307-11. Epub 2010/09/28.

6. Moayyeri A, Luben RN, Bingham SA, Welch AA, Wareham NJ, Khaw KT. Measured height loss predicts fractures in middle-aged and older men and women: the EPIC-Norfolk prospective population study. Journal of bone and mineral research : the official journal of the American Society for Bone and Mineral Research. Mar 2008;23(3):425-32. Epub 2007/11/14.

7. Briot K, Legrand E, Pouchain D, Monnier S, Roux C. Accuracy of patient-reported height loss and risk factors for height loss among postmenopausal women. CMAJ : Canadian Medical Association journal = journal de l'Association medicale canadienne. Apr 6 2010;182(6):558-62. Epub 2010/03/24.

8. Yoshimura N, Kinoshita H, Takijiri T, Oka H, Muraki S, Mabuchi A, et al. Association between height loss and bone loss, cumulative incidence of vertebral fractures and future quality of life: the Miyama study. Osteoporosis international : a journal established as result of cooperation between the European Foundation for Osteoporosis and the National Osteoporosis Foundation of the USA. Jan 2008;19(1):21-8. Epub 2007/10/27.

9. Tobias JH, Hutchinson AP, Hunt LP, McCloskey EV, Stone MD, Martin JC, et al. Use of clinical risk factors to identify postmenopausal women with vertebral fractures. Osteoporosis international : a journal established as result of cooperation between the European Foundation for Osteoporosis and the National Osteoporosis Foundation of the USA. Jan 2007;18(1):35-43. Epub 2006/09/05.

10. Krege JH, Siminoski K, Adachi JD, Misurski DA, Chen P. A simple method for determining the probability a new vertebral fracture is present in postmenopausal women with osteoporosis. Osteoporosis international : a journal established as result of cooperation between the European Foundation for Osteoporosis and the National Osteoporosis Foundation of the USA. 2006;17(3):379-86. Epub 2005/12/06.

11. Siminoski K, Jiang G, Adachi JD, Hanley DA, Cline G, Ioannidis G, et al. Accuracy of height loss during prospective monitoring for detection of incident vertebral fractures. Osteoporosis international : a journal established as result of cooperation between the European Foundation for Osteoporosis and the National Osteoporosis Foundation of the USA. Apr 2005;16(4):403-10. Epub 2004/08/17.

12. Ismail AA, Cooper C, Felsenberg D, Varlow J, Kanis JA, Silman AJ, et al. Number and type of vertebral deformities: epidemiological characteristics and relation to back pain and height loss. European Vertebral Osteoporosis Study Group. Osteoporosis international : a journal established as result of cooperation between the European Foundation for Osteoporosis and the National Osteoporosis Foundation of the USA. 1999;9(3):206-13. Epub 1999/08/18.

13. Huang C, Ross PD, Lydick E, Davis JW, Wasnich RD. Contributions of vertebral fractures to stature loss among elderly Japanese-American women in Hawaii. Journal of bone and mineral research : the official journal of the American Society for Bone and Mineral Research. Mar 1996;11(3):408-11. Epub 1996/03/01.

14. Jaturapatporn D, Hathirat S, Manataweewat B, Dellow AC, Leelaharattanarak S, Sirimothya S, et al. Reliability and validity of a Thai version of the General Practice Assessment Questionnaire (GPAQ). Journal of the Medical Association of Thailand = Chotmaihet thangphaet. Sep 2006;89(9):1491-6. Epub 2006/11/15.

15. Wolever TM, Chiasson JL, Csima A, Hunt JA, Palmason C, Ross SA, et al. Variation of postprandial plasma glucose, palatability, and symptoms associated with a standardized mixed test meal versus 75 g oral glucose. Diabetes care. Mar 1998;21(3):336-40. Epub 1998/04/16.

16. Levy JC, Matthews DR, Hermans MP. Correct homeostasis model assessment (HOMA) evaluation uses the computer program. Diabetes care. Dec 1998;21(12):2191-2. Epub 1998/12/05.

17. Levey AS, Stevens LA, Schmid CH, Zhang YL, Castro AF, 3rd, Feldman HI, et al. A new equation to estimate glomerular filtration rate. Annals of internal medicine. May 5 2009;150(9):604-12. Epub 2009/05/06.

18. Wang T, Li M, Chen B, Xu M, Xu Y, Huang Y, et al. Urinary bisphenol A (BPA) concentration associates with obesity and insulin resistance. The Journal of clinical endocrinology and metabolism. Feb 2012;97(2):E223-7. Epub 2011/11/18.

19. Executive Summary of The Third Report of The National Cholesterol Education Program (NCEP) Expert Panel on Detection, Evaluation, And Treatment of High Blood Cholesterol In Adults (Adult Treatment Panel III). Jama. May 16 2001;285(19):2486-97. Epub 2001/05/23.

20. Appropriate body-mass index for Asian populations and its implications for policy and intervention strategies. Lancet (London, England). Jan 10 2004;363(9403):157-63. Epub 2004/01/17.

21. Alberti KG, Zimmet P, Shaw J. The metabolic syndrome--a new worldwide definition. Lancet (London, England). Sep 24-30 2005;366(9491):1059-62. Epub 2005/09/27.

22. Levey AS, Coresh J, Balk E, Kausz AT, Levin A, Steffes MW, et al. National Kidney Foundation practice guidelines for chronic kidney disease: evaluation, classification, and stratification. Annals of internal medicine. Jul 15 2003;139(2):137-47. Epub 2003/07/16.

SUPPLEMENTAL ETABLES

| **Supplemental Table1-Baseline Characteristics for subjects with and without retinopathy** | | | |
| --- | --- | --- | --- |
|  | **Without Retinopathy** | **With Retinopathy** | **P Value** |
| n of cases | 8175 | 369 |  |
| Age (years) | 52 (46 to 60) | 62 (56 to 68) | 0.001 |
| Sex |  |  | 0.001 |
| <65 Men | 2911 (35.6) | 87 (23.6) |  |
| ≧65 Men | 666 (8.1) | 71 (19.2) |  |
| Premenopausal Women | 2147 (26.3) | 29 (7.9) |  |
| Postmenopausal Women | 2451 (30.0) | 182 (49.3) |  |
| High school or more | 3056 (37.4) | 124 (33.6) | **0.142** |
| Smokers | 1737 (21.2) | 111 (30.1) | 0.001 |
| Alcohol drinkers | 2394 (29.3) | 103 (27.9) | **0.571** |
| Coffee drinkers | 513 (6.3) | 12 (3.3) | 0.018 |
| Milk drinkers | 3888 (47.6) | 166 (45.0) | **0.333** |
| Bean products | 6480 (79.3) | 273 (74.0) | 0.015 |
| Physical activity |  |  | 0.001 |
| Low | 6143 (75.1) | 325 (88.1) |  |
| Moderate | 652 (8.0) | 19 (5.1) |  |
| High | 1380 (16.9) | 25 (6.8) |  |
| FBG (mmol/l) | 5.4 (5.1 to 5.9) | 6.2 (5.5 to 8.2) | 0.001 |
| PBG (mmol/l) | 6.6 (5.5 to 8.2) | 10.4 (7.1 to 15.7) | 0.001 |
| HbA1c (%) | 5.7 (5.4 to 6.0) | 6.2 (5.7 to 7.6) | 0.001 |
| FIN (μU/ml) | 5.9 (4.1 to 8.4) | 7.0 (4.8 to 10.8) | 0.001 |
| HOMA-IR | 1.4 (0.9 to 2.1) | 2.2 (1.4 to 3.3) | 0.001 |
| SBP (mmHg) | 131 (119 to 145) | 146 (133 to 161) | 0.001 |
| DBP (mmHg) | 77 (70 to 84) | 81 (73 to 89) | 0.001 |
| HDL (mmol/l) | 1.36 (1.16 to 1.58) | 1.32 (1.13 to 1.55) | 0.034 |
| LDL (mmol/l) | 2.95 (2.44 to 3.51) | 3.01 (2.49 to 3.60) | **0.314** |
| TC (mmol/l) | 5.12 (4.48 to 5.81) | 5.26 (4.68 to 5.92) | 0.006 |
| TG (mmol/l) | 1.31 (0.94 to 1.95) | 1.62 (1.17 to 2.39) | 0.001 |
| BMI(kg/㎡) | 23.9 (21.8 to 26.0) | 25.0 (23.0 to 27.3) | 0.001 |
| WHR | 0.87 (0.83 to 0.91) | 0.91 (0.87 to 0.94) | 0.001 |
| eGFR (ml/min/1.73㎡) | 96.6 (87.5 to 104.3) | 87.2 (74.0 to 94.6) | 0.001 |
| Diabetes | 1130 (13.8) | 211 (57.2) | 0.001 |
| IR | 1336 (16.3) | 154 (41.7) | 0.001 |
| Hypertension | 3275 (40.1) | 291 (78.9) | 0.001 |
| Dyslipidemia | 3027 (37.0) | 173 (46.9) | 0.001 |
| General obesity | 1139 (13.9) | 90 (24.4) | 0.001 |
| Abdominal obesity | 2808 (34.3) | 201 (54.5) | 0.001 |
| eGFR <60ml/min/1.73㎡ | 164 (2.0) | 30 (8.1) | 0.001 |
| Gastrointestinal disorders | 982 (12.0) | 36 (9.8) | **0.191** |
| Respiratory diseases | 195 (2.4) | 10 (2.7) | **0.690** |
| Urological diseases | 434 (5.3) | 24 (6.5) | **0.319** |
| T-score | 0.0 (﹣1.1 to 1.1) | ﹣0.6 (﹣1.6 to 0.4) | 0.001 |
| BMD Status |  |  | 0.001 |
| Normal BMD | 5853 (71.6) | 217 (58.8) |  |
| Low BMD | 2322 (28.4) | 152 (41.2) |  |
| Osteopenia | 2008 (24.6) | 121 (32.8) |  |
| Osteoporosis | 314 (3.8) | 31 (8.4) |  |
| Data are median (IQR) for continuous variables and count (%) for categorical variables. The difference among groups was examined by the Mann-Whitney test or Chi-squared test where appropriate. | | | |
| The bolded stands for no statistically significant (P≧0.05). | | | |
| **Abbreviations:** FBG, fasting blood glucose; PBG, postprandial blood glucose; HbA1c, glycated hemoglobin A1c; FIN, fasting serum insulin; HOMA-IR, homeostasis model assessment of insulin resistance; SBP, systolic blood pressure; DBP, diastolic blood triglycerides; BMI, body mass index; WHR, waist-to-hip ratio; eGFR, estimated glomerular filtration rate; IR, insulin resistance; | | | |

| **Supplemental Table2-1 Baseline and follow-up Characteristics for followed subjects with and without retinopathy in men** | | | | | | |
| --- | --- | --- | --- | --- | --- | --- |
|  | **<65 Men** **Retinopathy** | | **P Value** | **>65 Men Retinopathy** | | **P Value** |
|  | **None** | **Present** |  | **None** | **Present** |  |
| **Baseline Characteristics** |  |  |  |  |  |  |
| n of cases | 1888 | 57 |  | 268 | 38 |  |
| Age (years) | 49 (45 to 56) | 54 (49 to 58) | **0.001** | 68 (66 to 71) | 68(66 to 72) | 0.784 |
| High school or more | 853 (45.2) | 30 (52.6) | 0.266 | 96 (35.8) | 18 (47.4) | 0.168 |
| Smokers | 1061 (56.2) | 31 (54.4) | 0.444 | 124 (46.3) | 11 (28.9) | **0. 032** |
| Alcohol drinkers | 779 (41.3) | 23 (40.4) | 0.891 | 93 (34.7) | 11 (28.9) | 0.483 |
| Coffee drinkers | 107 (5.7) | 2 (3.5) | 0.685 | 8 (3) | 1 (2.6) | 0.999 |
| Milk drinkers | 970 (51.3) | 22 (38.6) | 0.057 | 142 (53.0) | 22 (57.9) | 0.570 |
| Bean products | 1481 (78.4) | 40 (70.2) | 0.136 | 211 (78.7) | 28 (73.7) | 0.481 |
| Physical activity |  |  | 0.128 |  |  | **0.013** |
| Low | 1186 (62.8) | 38 (66.7) |  | 242 (90.3) | 34 (89.5) |  |
| Moderate | 215 (11.4) | 10 (17.5) |  | 2(0.3) | 2(5.3) |  |
| High | 487 (25.8) | 9 (15.8) |  | 15 (9.3) | 2(5.3) |  |
| Diabetes (n [%]) | 254 (13.4) | 32 (56.1) | **0.001** | 61 (22.8) | 18 (47.4) | **0.002** |
| IR | 256 (13.6) | 26 (45.6) | **0.001** | 42 (15.7) | 16 (42.1) | **0.001** |
| Hypertension | 748 (39.6) | 43 (75.4) | **0.001** | 176 (65.7) | 35 (92.1) | **0.001** |
| Dyslipidemia | 790 (41.8) | 35 (61.4) | **0.004** | 100 (37.3) | 18 (47.4) | 0.285 |
| General obesity | 292 (25.4) | 20 (35.1) | **0.001** | 43 (16) | 12 (31) | **0.02**0 |
| Abdominal obesity | 395 (20.9) | 25 (43.9) | **0.001** | 69 (25.7) | 19 (50.0) | **0.002** |
| eGFR <60ml/min/1.73 ㎡ | 24 (1.3) | 3 (5.3) | **0.050** | 16 (6) | 6 (15.8) | 0.063 |
| Gastrointestinal disorders | 227 (12) | 6 (10.5) | 0.732 | 34 (12.7) | 6 (15.8) | 0.607 |
| Respiratory diseases | 29 (1.5) | 0 (0) | 0.698 | 15 (5.6) | 2 (5.3) | 0.999 |
| Urological diseases | 110 (5.8) | 7 (12.3) | 0.082 | 16 (6) | 3 (7.9) | 0.920 |
| BMD Status |  |  | **0.004** |  |  | 0.428 |
| Normal BMD | 1401 (74.4) | 34 (59.6) |  | 166 (61.9) | 22 (57.9) |  |
| Low BMD | 485 (25.6) | 23 (40.4) |  | 102 (38.1) | 16 (42.1) |  |
| Osteopenia | 436 (23.1) | 18 (31.6) |  | 88 (32.8) | 12 (31.6) |  |
| Osteoporosis | 48 (2.5) | 5 (8.8) |  | 14 (5.2) | 4 (10.5) |  |
| **Follow-up changing** |  |  |  |  |  |  |
| Height loss at least 2 cm | 281 (14.9) | 13 (22.8) | 0.100 | 64 (23.9) | 15 (39.5) | **0.040** |
| New Smokers | 136 (7.2) | 1 (1.8) | 0.186 | 23 (8.6) | 6 (15.8) | 0.261 |
| New alcohol drinkers | 161 (8.5) | 7 (12.3) | 0.451 | 23 (8.6) | 4 (10.5) | 0.928 |
| New diabetes | 115 (6.1) | 1 (1.8) | 0.281 | 20 (7.5) | 2 (5.3) | 0.876 |
| New hypertension | 204 (10.8) | 4 (7) | 0.362 | 29 (10.8) | 0 (0) | 0.066 |
| New dyslipidemia | 299 (15.8) | 8 (14) | 0.713 | 41 (15.1) | 4 (10.5) | 0.437 |
| New gastrointestinal disorders | 88 (4.7) | 3 (5.3) | 0.999 | 6 (2.2) | 1 (2.6) | 0.999 |
| New respiratory diseases | 23 (1.2) | 1 (1.8) | 0.512 | 7 (2.6) | 2 (5.3) | 0.999 |
| New urological diseases | 75 (4) | 5 (8.8) | 0.145 | 23 (8.6) | 3 (7.9) | 0.999 |
| General obesity changed | 106 (5.6) | 9 (15.8) | **0.003** | 19 (7.1) | 5 (13.2) | 0.327 |
| Abdominal obesity changed | 322 (17.0) | 12 (21.1) | 0.424 | 65 (24.3) | 9 (23.7) | 0.939 |
| eGFR<60ml/min/1.73m^2^ changed | 21 (1.1) | 2 (3.5) | 0.144 | 15 (5.6) | 5 (13.2) | 0.078 |
| Data are median (IQR) for continuous variables and count (%) for categorical variables. The difference among groups was examined by theMann-Whitney test or Chi-squared test where appropriate. The bolded stands for no statistically significant (P>0.05) | | | | | | |
| **Abbreviations:** BMI, bodymass index; eGFR, estimated glomerular filtration rate; IR, insulin resistance; | | | | | | |

| **Supplemental Table2-2 Baseline and follow-up Characteristics for followed subjects with and without** **retinopathy in women** | | | | | | |
| --- | --- | --- | --- | --- | --- | --- |
|  | **Premenopausal Women** **Retinopathy** | | **P Value** | **Postmenopausal Women Retinopathy** | | **P Value** |
|  | **None** | **Present** |  | **None** | **Present** |  |
| **Baseline Characteristics** |  |  |  |  |  |  |
| n of cases | 1267 | 9 |  | 1409 | 122 |  |
| Age (years) | 45 (42 to 48) | 47 (43 to 51) | 0.183 | 57 (53 to 62) | 62 (57 to 67) | **0.001** |
| High school or more | 545 (43.6) | 2 (22.2) | 0.359 | 401 (28.3) | 26 (21.1) | 0.091 |
| Smokers | 1 (0.1) | 0 (0) | 0.999 | 11 (0.8) | 2 (1.6) | 0.278 |
| Alcohol drinkers | 53 (4.2) | 0 (0) | 0.999 | 73 (5.1) | 5 (4.1) | 0.624 |
| Coffee drinkers | 111 (8.8) | 0 (0) | 0.999 | 63 (4.5) | 6 (4.9) | 0.819 |
| Milk drinkers | 593 (46.8) | 3 (33.3) | 0.515 | 606 (43.0) | 43 (35.2) | 0.096 |
| Bean products | 1036 (81.8) | 6 (66.7) | 0.463 | 1094 (77.6) | 88 (72.1) | 0.164 |
| Physical activity |  |  | 0.540 |  |  | **0.047** |
| Low | 966 (76.2) | 8 (88.9) |  | 1268 (90.0) | 119 (96.7) |  |
| Moderate | 155 (12.2) | 1 (11.1) |  | 46 (3.3) | 2 (1.6) |  |
| High | 146 (11.5) | 0 (0) |  | 95 (6.7) | 2 (1.6) |  |
| Diabetes | 79 (6.2) | 6 (66.7) | **0.001** | 248 (17.6) | 63 (51.6) | **0.001** |
| IR | 177 (14) | 4 (44.4) | **0.033** | 321 (22.8) | 45 (36.9) | **0.001** |
| Hypertension | 276 (21.8) | 8 (88.9) | **0.001** | 702 (49.8) | 93 (76.2) | **0.001** |
| Dyslipidemia | 301 (23.8) | 3 (33.3) | 0.452 | 622 (44.1) | 60 (49.2) | 0.297 |
| General obesity | 135 (10.7) | 3 (33.3) | 0.064 | 249 (17.5) | 30 (24.4) | 0.059 |
| Abdominal obesity | 424 (33.5) | 7 (77.8) | **0.005** | 746(52.9) | 84 (68.0) | **0.001** |
| eGFR <60ml/min/1.73 ㎡ | 6 (0.5) | 0 (0.0) | 0.999 | 34 (2.4) | 8 (6.6) | **0.016** |
| Gastrointestinal disorders | 106 (8.4) | 0 (0) | 0.999 | 182 (12.9) | 9 (7.4) | 0.072 |
| Respiratory diseases | 20 (1.6) | 0 (0) | 0.999 | 19 (1.3) | 2 (1.6) | 0.999 |
| Urological diseases | 47 (3.7) | 0 (0) | 0.999 | 64 (4.5) | 7 (5.7) | 0.547 |
| BMD Status |  |  | 0.686 |  |  | 0.277 |
| Normal BMD | 1169 (92.13) | 9 (100) |  | 907 (64.4) | 74 (60.7) |  |
| Low BMD | 98 (7.7) | 0 (0) |  | 505 (35.6) | 49 (39.3) |  |
| Osteopenia | 94 (7.4) | 0 (0) |  | 415 (29.5) | 36 (29.5) |  |
| Osteoporosis | 4 (0.3) | 0 (0) |  | 87 (6.2) | 12 (9.8) |  |
| **Follow-up changing** |  |  |  |  |  |  |
| Height loss at least 2 cm | 197 (15.5) | 2 (22.2) | 0.929 | 322 (22.9) | 30 (24.6) | 0.662 |
| New Smokers | 1 (0.1) | 0 (0) | 0.999 | 5 (0.4) | 2 (1.6) | 0.102 |
| New alcohol drinkers | 15 (1.2) | 0 (0) | 0.999 | 26 (1.8) | 2 (1.6) | 0.999 |
| New diabetes | 62 (4.9) | 0 (0) | 0.999 | 114 (8.1) | 6 (4.9) | 0.211 |
| New hypertension | 122 (9.6) | 1 (11.1) | 0.600 | 165 (11.7) | 12 (9.8) | 0.535 |
| New dyslipidemia | 226 (17.8) | 2 (22.2) | 0.999 | 247 (17.4) | 15 (12.2) | 0.141 |
| New gastrointestinal disorders | 50 (3.9) | 1 (11.1) | 0.308 | 58 (4.1) | 4 (3.3) | 0.833 |
| New respiratory diseases | 3 (0.2) | 0 (0.0) | 0.999 | 10 (0.7) | 1 (0.8) | 0.600 |
| New urological diseases | 33 (2.6) | 0 (0) | 0.999 | 42 (3.0) | 5 (4.1) | 0.680 |
| General obesity changed | 57 (4.5) | 1 (11.1) | 0.343 | 105 (7.5) | 13 (10.7) | 0.203 |
| Abdominal obesity changed | 321 (17.0) | 0 (0.0) | 0.174 | 366 (26.0) | 23(18.9) | 0.083 |
| eGFR<60ml/min/1.73m^2^ changed | 7 (0.6) | 0 (0) | 0.999 | 41 (2.9) | 12 (9.8) | **0.001** |
| Data are median (IQR) for continuous variables and count (%) for categorical variables. The difference among groups was examined by theMann-Whitney test or Chi-squared test where appropriate. The bolded stands for statistically significant (P<0.05) | | | | | | |
| **Abbreviations:** BMI, bodymass index; eGFR, estimated glomerular filtration rate; IR, insulin resistance; | | | | | | |
